# A Comparative Evaluation of GPT-4 Turbo and Gemini-Pro in Medical Licensing Exams: Enhancing Artificial Intelligence's Role in Medical Education

**DOI:** 10.7759/cureus.101101

**Published:** 2026-01-08

**Authors:** Tom T Sussan, Rami J Sussan, Adli G Atkinson, Isaac H Atkinson, Kayle Cunningham, Joshua Eckroth, Leonard B Miller, Tao Wei

**Affiliations:** 1 Department of Computer Science, Stetson University, Deland, USA; 2 Medical Education, Lake Erie College of Osteopathic Medicine, Bradenton, USA; 3 Cardiology, Florida International University, Miami, USA; 4 Department of Plastic and Reconstructive Surgery, Harvard Medical School, Boston, USA; 5 College of Medicine, Lake Erie College of Osteopathic Medicine, Bradenton, USA

**Keywords:** artificial intelligence and education, board exam, chatgpt, chatgpt 4, medical education technology, openai, preclinical education, usmle exams, usmle-style multiple-choice questions

## Abstract

Background and objective

Large language models (LLMs) are increasingly being explored as adjuncts to medical education; however, comparative data on the performance and error patterns of newer models on standardized licensing-style questions remain limited. This study evaluated two advanced large language models (LLMs) - Gemini-Pro and GPT-4 Turbo - on the National Board of Medical Examiners (NBME) Step 1-style multiple-choice questions to assess accuracy, reasoning quality, and common failure modes relevant to exam preparation and clinical reasoning training.

Methods

A total of 112 NBME Step 1 questions were collected; seven image- or table-dependent items were excluded, yielding 105 text-only questions. Prompts were standardized to include the clinical stem, query, and answer choices and were submitted via Python API to Gemini-Pro and GPT-4 Turbo. Outputs were independently adjudicated by two third-year medical students and one board-certified physician using binary accuracy scoring and structured evaluation of reasoning features (logical reasoning, internal information use, and external knowledge application). Incorrect responses were categorized as logical, informational, or statistical errors. Comparative analyses included raw accuracy calculations and chi-square testing of reasoning-feature distributions.

Results

GPT-4 Turbo achieved 90.99% accuracy on the January 2024 NBME Step 1 question set, substantially outperforming Gemini-Pro (54.46%). GPT-4 Turbo demonstrated fewer errors overall, with lower logical (16%), informational (4%), and statistical (4%) error rates compared with older baselines reported in the study (e.g., GPT-3.5 logical errors 42%). GPT-4 Turbo incorporated external information in 76% of correct responses versus 25% for Gemini-Pro, and differences in performance metrics between GPT-4 Turbo and Gemini-Pro were statistically significant (p < 0.05).

Conclusions

GPT-4 Turbo markedly outperformed Gemini-Pro on text-based NBME Step 1 questions, showing higher accuracy, stronger reasoning consistency, and fewer logical/informational failures. These findings support GPT-4 Turbo’s potential role as a high-yield supplementary tool for Step 1-style learning and feedback, while underscoring the need for continued refinement and cautious, supervised integration of LLMs into medical education, given persistent (though reduced) error rates.

## Introduction

Architecture

Large language models (LLMs) are a class of artificial intelligence (AI) systems built on neural networks that learn patterns in language and generate responses by predicting sequences of tokens. Vaswani et al. introduced the Transformer architecture, which uses self-attention to process tokens in parallel, enabling rapid contextual learning [1]. This allows each token to be evaluated in relation to every other token in the sequence, capturing dependencies more effectively than sequential models. Brakel et al. explained that neural networks within LLMs serve as artificial neurons, adjusting weights and biases to learn from data [2]. Aitken et al. detailed how the Transformer architecture consists of encoders and decoders, with the encoder establishing context and the decoder generating output based on both encoder data and previous tokens [3]. Within each encoder and decoder block, multi-head self-attention mechanisms assess the probability and contextual relevance of tokens. The Scaled Dot-Product attention mechanism mathematically weighs these relationships, enabling nuanced language understanding.

Training mechanism/genesis of response

LLMs are trained on large datasets from the internet, including articles, websites, and e-books. Tokenization is the first step in processing, breaking text into smaller units. Zouhar et al. described Byte-Pair Encoding (BPE) as a common tokenization method that iteratively merges frequent character pairs [4], while Choo and Kim examined SentencePiece, which uses probabilistic subword segmentation [5]. The training process assigns numerical IDs to tokens, allowing neural networks to model the probability of each token’s occurrence in a sequence. Models typically use autoregressive language modeling, predicting the next token step-by-step. Xu et al. noted that cross-entropy loss is the standard measure for assessing prediction accuracy during training [6]. Lin et al. proposed more data-efficient fine-tuning strategies, combining supervised fine-tuning with reinforcement learning from human feedback to improve task-specific performance [7]. Despite training, most LLMs lack persistent memory beyond a session, relying heavily on immediate context.

Model reconstruction

The LLM learns from the reaction to its response. Perplexity, accuracy, and precision are metrics by which the program measures its performance. Perplexity is the inverse probability of the response, normalized by the number of words in the response. A lower perplexity score suggests that the model accumulated a higher probability response. Accuracy measures how often the program is right, while precision measures the proportion of similar true outputs compared to all actual outputs.

AI in healthcare and biomedical research

Artificial intelligence has been investigated in medicine for decades, with foundational work describing clinical relevance well before the current LLM era. Ramesh et al. described AI’s role in medicine as early as 2004 [8]. Jiang et al. reviewed AI’s presence in healthcare, noting applications in clinical, pharmaceutical, managerial, and educational contexts [9]. During the SARS-CoV-2 pandemic, Coban et al. used in silico screening to analyze more than 30 million chemotypes, identifying 350 high-value antiviral candidates [10]. Such capacity illustrates AI’s potential to accelerate traditionally lengthy drug discovery processes. Shafqat et al. predicted U.S. health data could reach yottabytes (10^24 GB) [11], underscoring the need for systems capable of managing vast datasets.

Dixit et al. highlighted companies such as Deep Genomics, Grail, and PathAI that leverage AI to predict therapeutic outcomes and aid early cancer detection [12]. Griffin et al. described PathAI’s use of AI to analyze biopsy tissue [13], while Klein et al. validated Grail’s Galleri blood test for detecting up to 50 cancer types [14]. Amann et al. examined the challenge of integrating domain-specific medical data into AI due to privacy and interoperability barriers [15]. Despite these constraints, general-domain AI models have matched or exceeded domain-specific systems in certain imaging applications, such as dermatology (Li et al. [16]) and ophthalmology (Ting et al. [17]).

AI adoption and expansion into medical education

As general-purpose LLMs became widely available, interest surged in their potential role in education-especially in high-stakes professional training. Wu et al. found that merely 60 days after its release, ChatGPT was engaging over 100 million users monthly [18], reflecting not only public interest but the potential for AI to enter domains such as medicine, where it has been investigated for over a decade. In medical licensing exam contexts, early evaluations demonstrated moderate baseline performance with rapid improvement across iterations. In 2021, Kung et al. assessed GPT-3 on the United States Medical Licensing Examination (USMLE) and reported a Step 1 accuracy of 45.4% without prompting [19]. Burk-Rafel et al. estimated that students study 300-400 hours for this exam [20]. Gilson et al. later showed ChatGPT-3.5 achieving 64.4% accuracy [21]. These trends motivated further study into whether newer models can enhance problem-solving, critical thinking, and knowledge application in medical education.

ChatGPT vs. Gemini

With multiple widely used LLM platforms now available, comparing their architecture and performance characteristics has become increasingly relevant. Islam and Ahmed reported that both ChatGPT and Gemini use decoder-only Transformer architectures [22]. Rane et al. explained that Gemini’s training is based on Google’s curated datasets, whereas ChatGPT’s sources are broader and more variable [23]. Kishore and Shaik compared the two, finding ChatGPT better at conversational nuance while Gemini exhibited a more extensive vocabulary range [24].

ChatGPT 3.5 operated on 175 billion parameters, and the number of parameters for its newest model, GPT-4, is undisclosed. Additional LLMs, such as Google’s AI language model of Gemini, have recently surfaced as widely used platforms. These technologies, demonstrating an ability to critically analyze vast amounts of information in ways scarcely seen before, illustrate the proximity of AI penetration into clinical care and medical education. As rapidly evolving products, the AI platforms should be closely monitored. This study seeks to evaluate the newest product versions of ChatGPT and Gemini, GPT-4 and Gemini Pro, in their ability to perform across the USMLE Step 1.

Comparison of human and chatbot performance

Peer-reviewed evaluations indicate that large language model-based chatbots can achieve examination-level performance comparable to that of medical students on standardized medical licensing assessments. Initial studies demonstrated that ChatGPT, based on GPT-3.5, performed at or near the minimum passing threshold across all three steps of the United States Medical Licensing Examination (USMLE), establishing baseline competence in factual recall and structured clinical reasoning without medical fine-tuning. [19]. Subsequent investigations of more advanced systems, including GPT-4 and GPT-4o, showed substantial performance gains, with these models achieving passing or above-passing accuracy and, in controlled settings, exceeding the average performance of medical student cohorts on identical multiple-choice and vignette-based examinations [25].

Medically optimized models such as Med-PaLM and Med-PaLM 2 further improved performance on medical knowledge benchmarks, approaching expert-level results on selected tasks [26]. Despite these advances, resident physicians and attending clinicians consistently outperform current large language models in domains requiring contextual judgment, integration of longitudinal patient information, and real-world clinical decision-making. Collectively, these findings support the use of standardized examinations as reproducible benchmarks for evaluating chatbot capabilities while underscoring that examination performance alone should not be interpreted as equivalent to clinical competence.

## Materials and methods

Medical education data source and preprocessing 

The questions were sourced from the National Board of Medical Examiners (NBME) Step 1 exam. A total of 112 questions were collected, focusing on text-based items that could be processed by large language models without visual input. Seven questions requiring image interpretation or table-based analysis were excluded to ensure compatibility with text-only processing.

The remaining questions were formatted to ensure consistency in the input structure. Each question prompt included the stem (the clinical scenario), followed by the query and the multiple-choice options. This standardized format was designed to normalize the way information was presented to Gemini, Gemini-Pro, ChatGPT-3.5, and ChatGPT-4 Turbo, facilitating a direct comparison of their responses.

Prompt engineering and logical structuring

**Figure 1:**
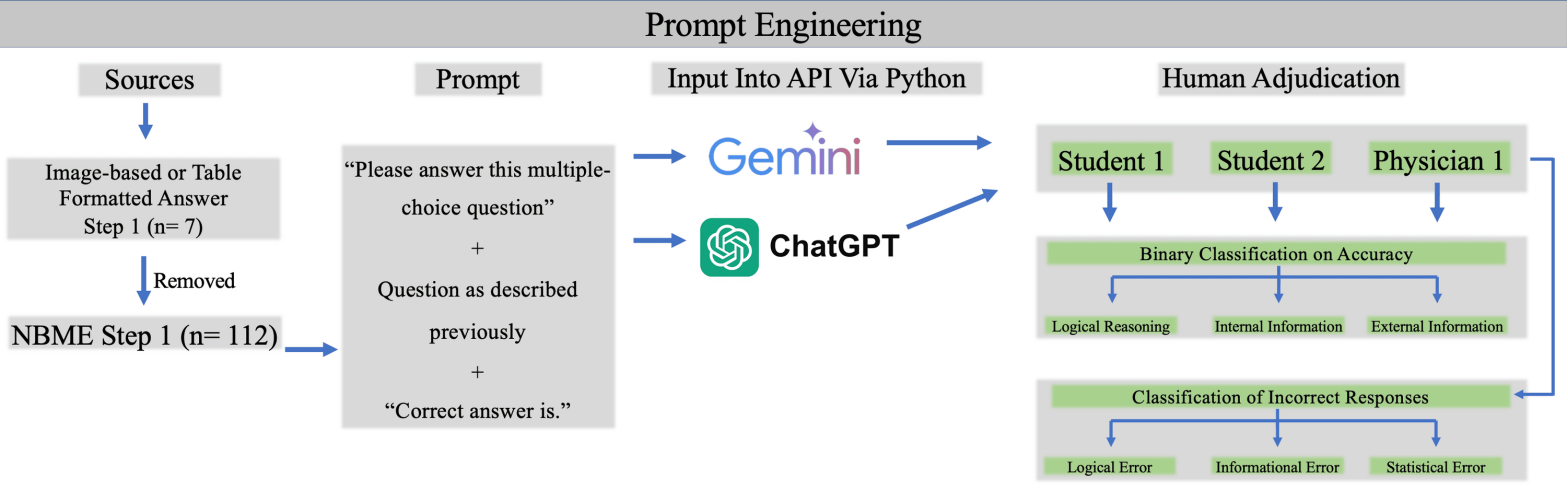
Schematic representation of the study design, beginning with NBME Step 1 questions (n = 112) after removal of image- and table-based items Standardized multiple-choice prompts were created and submitted via Python API to Gemini and ChatGPT. Responses were independently adjudicated by two third-year medical students and one board-certified physician, with binary classification for accuracy and categorization of errors into logical, informational, or statistical types NBME: National Board of Medical Examiners

Prompt engineering was an essential part of this study, aimed at getting Gemini, Gemini-Pro, GPT-3.5, and GPT-4 Turbo to provide the most accurate and well-reasoned answers (Figure 1). The structured prompts followed a uniform template that began with the clinical scenario, posed the query, and listed the multiple-choice options. Additionally, prompts were crafted to explicitly request the models to justify their answer choices, thereby encouraging the application of logical reasoning. The logical structuring of the prompts involved guiding the models to not only choose the correct answer but also to provide a rationale grounded in the medical information presented. This process aimed to assess the models' ability to integrate the contextual details of the question into their reasoning, which is crucial for accurate medical decision-making.

Response adjudication and evaluation criteria

The responses generated by the AI models were subjected to a rigorous adjudication process involving a trio of binary variables: logical reasoning, internal information, and external information. These variables were independently assessed by two third-year medical students, selected for their recent completion of NBME Step 1, and one board-certified clinical professor to ensure both relevance and adjudicative rigor.

Logical Reasoning

This criterion evaluated the AI models' ability to justify their answer choices based on the logical application of the information provided in the question stem. The models were expected to demonstrate a clear rationale that aligned with standard medical reasoning practices.

Internal Information

This criterion assessed the extent to which the AI models incorporated and reflected upon details intrinsic to the question. A strong performance in this area indicated the models' capacity to understand and utilize the specific medical context presented in the question.

External Information Application

This criterion examined the models' ability to enhance their responses by integrating relevant knowledge beyond the immediate scope of the question. This involved the application of general medical knowledge that could support or reinforce the chosen answer.

Error Classification and Analysis

In cases where the AI models produced incorrect responses, the errors were classified into three distinct types: logical errors, informational errors, and statistical errors.

Logical errors: These occurred when the models failed to apply relevant information correctly, resulting in an inappropriate or illogical recommendation. An example might be choosing an answer that contradicts well-established medical guidelines based on the provided information.

Informational errors: These were identified when the models overlooked critical data or lacked essential external knowledge necessary for making an accurate decision. This type of error often involves missing key clinical details that should have informed the correct answer.

Statistical errors: These errors encompassed inaccuracies in numerical reasoning or the misinterpretation of statistical data. This ranged from basic arithmetic mistakes to more complex misjudgments in data prevalence or statistical relevance.

The classification of these errors provided insights into the specific challenges each model faced, allowing for a detailed comparison of their strengths and weaknesses.

Implementation and coding methodology

The prompts were input into the AI models using Python through Microsoft Visual Studio Code, specifically leveraging the OpenAI and Google APIs to interact with GPT-4 Turbo and Gemini-Pro, respectively. The Python scripts were designed to automate the process of prompt submission, response collection, and preliminary categorization of the results.

Data from the AI outputs were systematically stored in a structured database, ensuring efficient and organized access for subsequent analysis. All analyses were performed using Python (version 3.11; Python Software Foundation). The raw accuracy of each model was calculated as the proportion of correct responses. Additionally, an unpaired chi-square test was employed to assess the distribution of logical reasoning, internal information, and external information between correct and incorrect responses, allowing for a comparative evaluation of the performance metrics across the different models.

## Results

**Figure 2:**
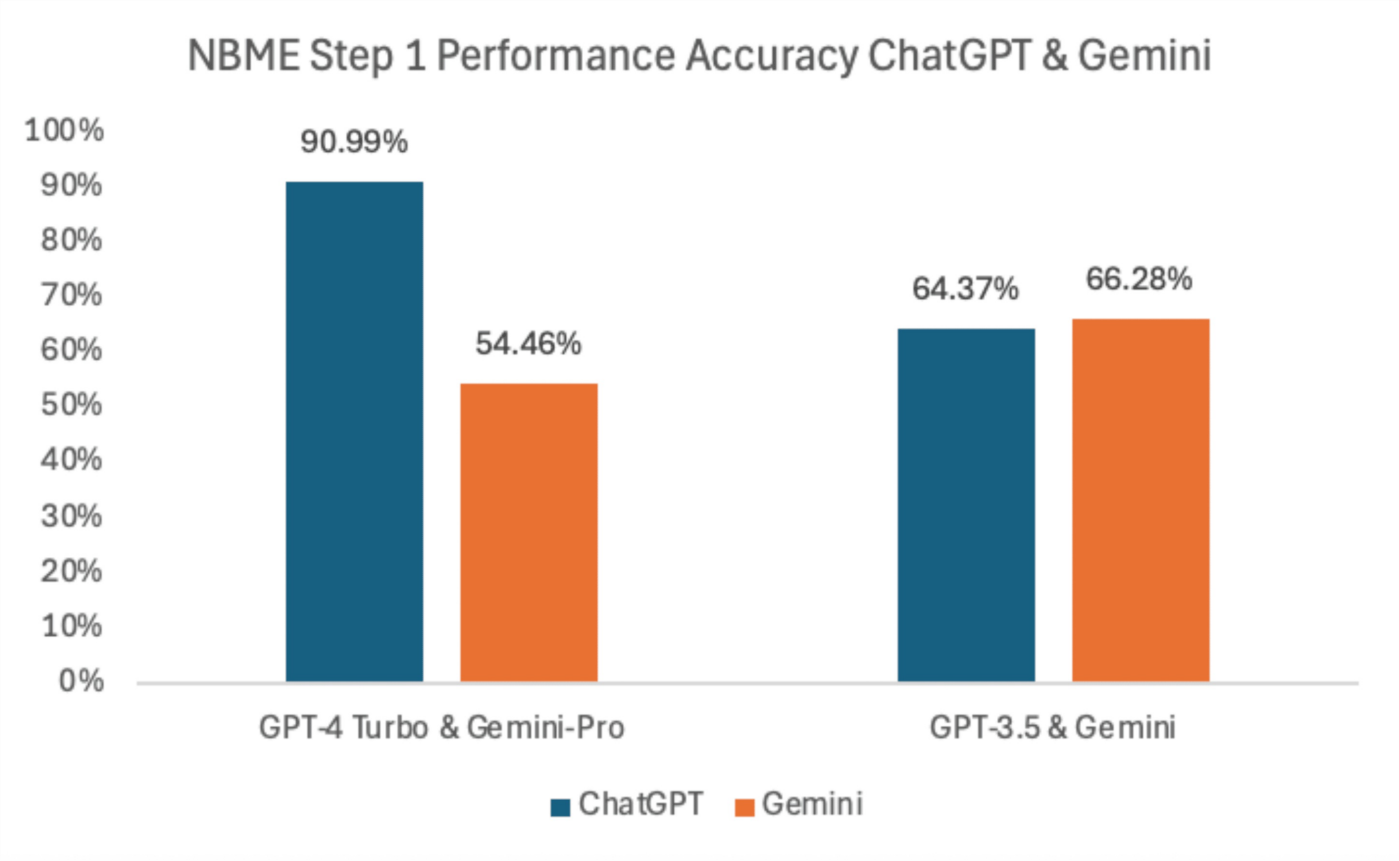
Comparative accuracy of ChatGPT and Gemini models on NBME Step 1 questions GPT-4 Turbo achieved a 90.99% accuracy rate on the January 2024 NBME Step 1 questions, substantially outperforming Gemini-Pro (54.46%). On the June 2022 NBME Step 1 questions, GPT-3.5 and Gemini demonstrated comparable performance, with accuracy rates of 64.37% and 66.28%, respectively NBME: National Board of Medical Examiners

GPT-4 Turbo achieved an accuracy rate of 90.99% on the January 2024 NBME Step 1 questions. Gemini-Pro, on the same set of questions, achieved an accuracy rate of 54.46% (Figure 2). The older models, GPT-3.5 and Gemini, had accuracy rates of 64.37% and 66.28% respectively, on the June 2022 NBME Step 1 questions.

Error classification

**Figure 3:**
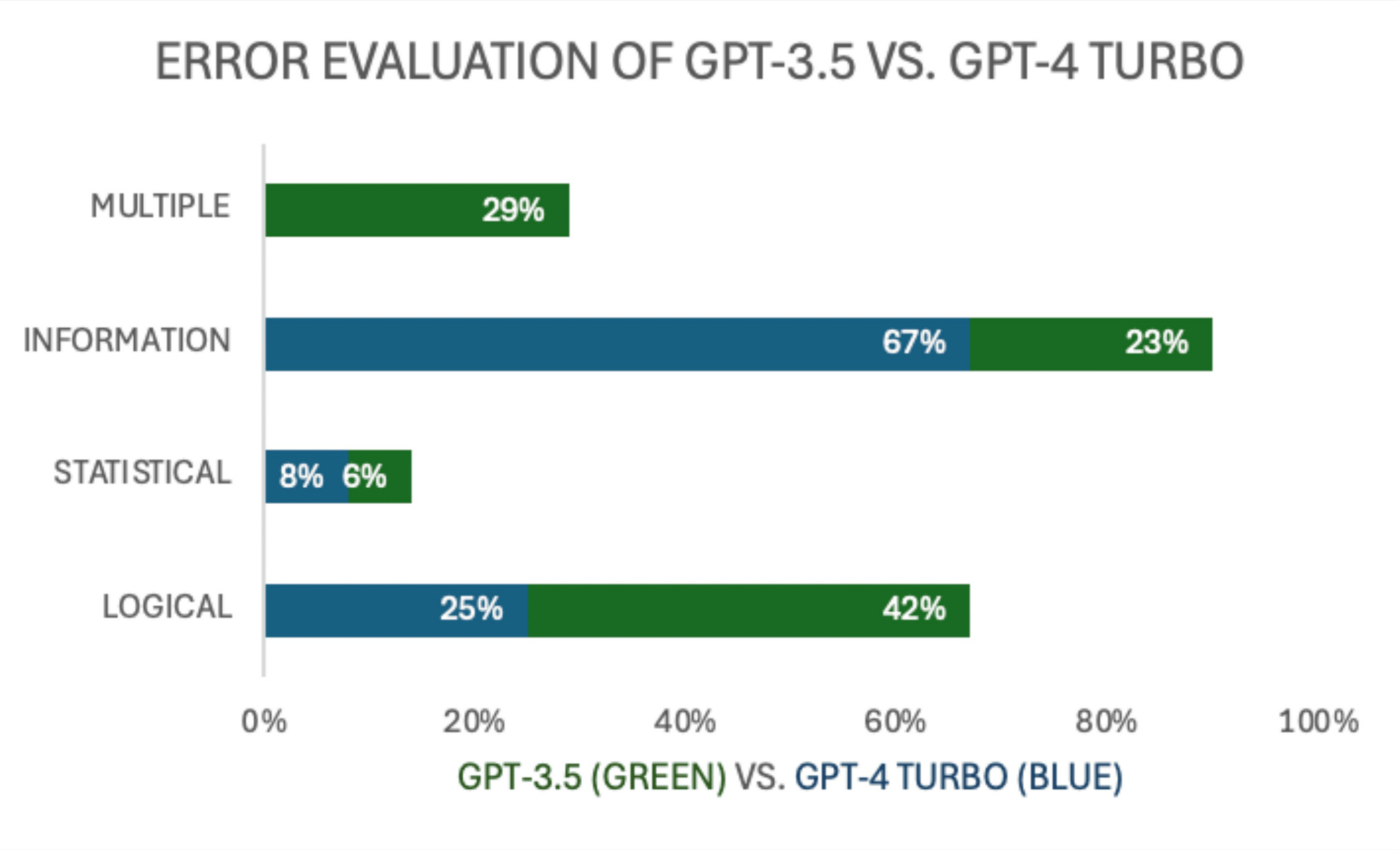
Comparative error analysis of ChatGPT-3.5 and GPT-4 Turbo on NBME Step 1 questions Bar chart displaying the percentage of multiple, informational, statistical, and logical errors for ChatGPT-3.5 (green) and GPT-4 Turbo (blue) NBME: National Board of Medical Examiners

GPT-4 Turbo demonstrated a significant reduction in logical errors compared to GPT-3.5, with a logical error rate of 16% compared to 42% for GPT-3.5. Informational errors were also reduced in GPT-4 Turbo, with only 4% of responses containing such errors, compared to 8% in GPT-3.5. Statistical errors were minimal across all models, with GPT-4 Turbo showing the lowest rate at 4%, compared to 6% in GPT-3.5 (Figure 3).

**Figure 4:**
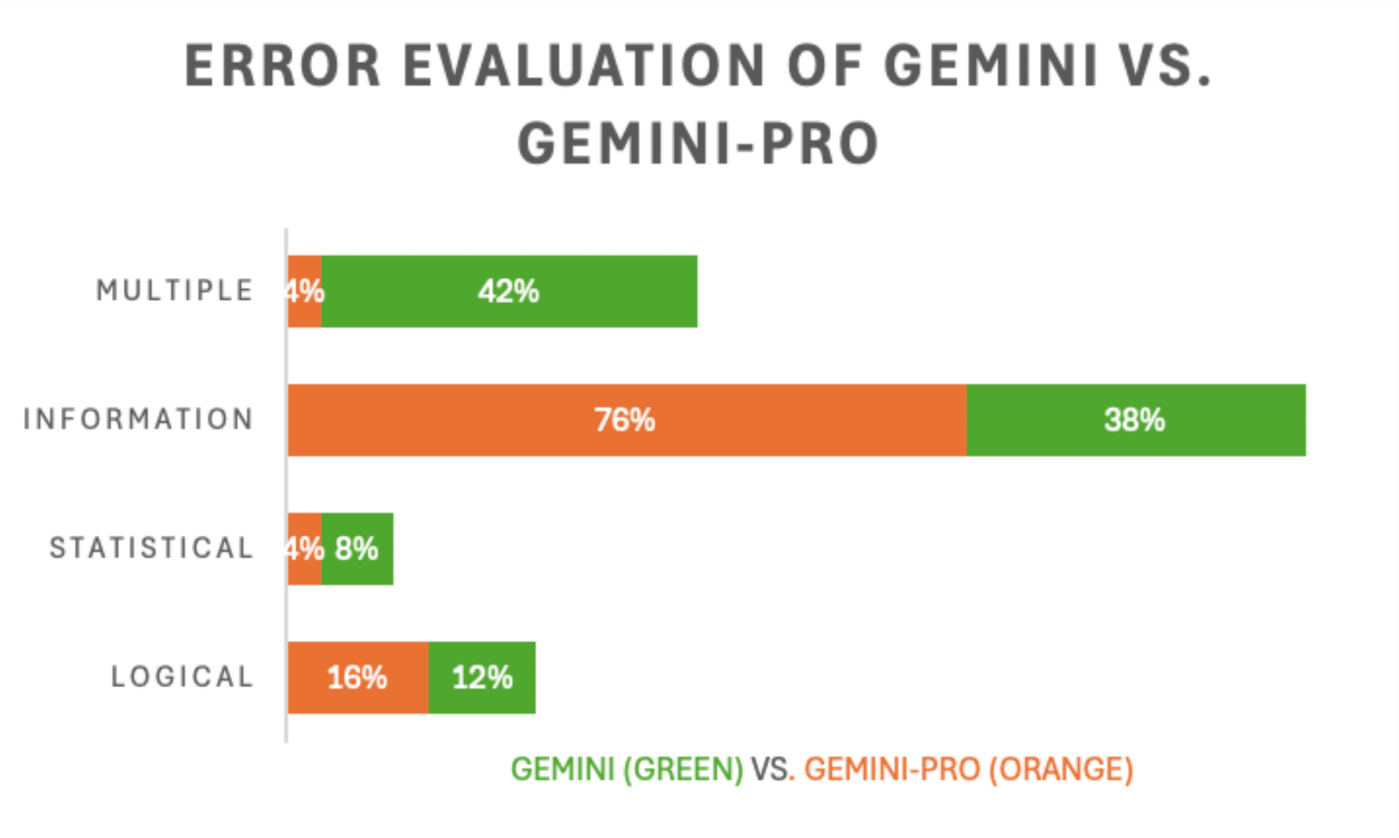
Comparative error analysis of Gemini and Gemini-Pro on NBME Step 1 questions Bar chart displaying the percentage of multiple, informational, statistical, and logical errors for Gemini (green) and Gemini-Pro (orange) NBME: National Board of Medical Examiners

Gemini-Pro, while improving upon some aspects of the older Gemini model, still exhibited a higher overall error rate (Figure 4). Logical errors in Gemini-Pro were recorded at 25%, with informational errors at 8%, both of which were higher than those observed in GPT-4 Turbo. The Gemini model displayed similar trends, with logical errors at 12% and informational errors at 8%. Errors in AI responses were classified into three categories: logical errors, informational errors, and statistical errors. GPT-4 Turbo exhibited a logical error rate of 16%, compared to 42% in GPT-3.5. Informational errors were recorded in 4% of GPT-4 Turbo’s responses, while GPT-3.5 had an informational error rate of 8%. Statistical errors were minimal across the models, with GPT-4 Turbo having a 4% statistical error rate, and GPT-3.5 at 6%. For Gemini-Pro, the logical error rate was 25%, with informational errors at 8%, and statistical errors at 4%. The Gemini model showed a logical error rate of 12% and an informational error rate of 8%.

Distribution of logical reasoning, internal information, and external information

The chi-square test revealed that GPT-4 Turbo consistently applied internal and external information more accurately than Gemini, Gemini-Pro, and GPT-3.5. For instance, GPT-4 Turbo incorporated external information in 76% of its correct responses, a notable improvement over Gemini-Pro, which did so in only 25% of its correct answers. GPT-4 Turbo also showed a strong performance, with logical reasoning applied correctly in 90.99% of the responses. This was significantly higher than the performance of Gemini-Pro, which correctly applied logical reasoning in 54.46% of its responses. The statistical analysis confirmed a significant difference in performance between the models, particularly between GPT-4 Turbo and Gemini-Pro (p < 0.05).

## Discussion

Principal findings

The results of this study demonstrate that GPT-4 Turbo significantly outperforms both Gemini-Pro and its predecessor GPT-3.5 in terms of accuracy and error reduction when tasked with answering NBME Step 1 questions. These questions GPT-4 Turbo's accuracy rate of 90.99% on the January 2024 NBME Step 1 questions positions it as a highly reliable tool for processing and applying medical knowledge, suggesting that this model has made substantial advancements in handling complex medical queries. Errors generated from incorrect answer responses were categorized into three sections: logical errors, informational errors, and statistical errors. GPT-4 Turbo outclassed GPT-3.5 in every category, providing more nuanced and contextually relevant responses. Gemini Pro exhibited higher logical and informational errors than GPT-4 Turbo; however, both LLMs had similar results in producing informational errors.

Gemini-Pro, while showing some improvements over the older Gemini model, still lags behind GPT-4 Turbo, particularly in its ability to minimize logical and informational errors. According to Rane et al., this may be due to Gemini’s limitation of only utilizing Google’s database, providing more “factual” responses compared to GPT’s pattern recognition [23]. This performance disparity highlights the importance of continuous refinement in AI models, especially when they are used in high-stakes environments like medical education and other fields alike. 

Implications for medical education

The superior performance of GPT-4 Turbo has important implications for its integration into medical education. Given its high accuracy and low error rates, GPT-4 Turbo could serve as a valuable tool for medical students, providing reliable feedback on complex medical queries. The ability of GPT-4 Turbo to consistently apply logical reasoning and incorporate both internal and external information into its responses suggests that it could enhance the learning experience by reinforcing critical thinking skills and deepening students' understanding of medical concepts. Furthermore, the model's adaptability to new data, as demonstrated by its performance on the January 2024 NBME Step 1 questions, proves its potential as a dynamic educational tool that can stay current with the latest medical knowledge. This adaptability is particularly crucial in the rapidly evolving field of medicine, where students must continuously update their knowledge base to keep pace with new developments.

In contrast, Gemini-Pro's lower accuracy and higher error rates indicate that while it may still be useful in educational settings, it requires further development before it can be relied upon as a primary resource. Lee et al. suggest, due to its current limitations, that it may be better suited for supplementary use, where its insights can be cross-verified with other sources to ensure accuracy [27]. While chatbots can efficiently generate multiple case-based questions, tutor oversight remains essential to ensure that assessments are clinically grounded and reflective of real-world practice rather than relying solely on AI-crafted scenarios.

Comparison with previous models and studies

The advancements seen in GPT-4 Turbo compared to GPT-3.5 and Gemini-Pro are consistent with trends observed in the development of large language models (LLMs). Gill et al. described these models as iteratively refined, tending to improve in their ability to handle complex reasoning tasks and apply domain-specific knowledge effectively [25]. In comparison to previous research, where earlier versions of AI models struggled with the generalizability and accuracy on medical exams, GPT-4 Turbo represents a significant leap forward. Its ability to outperform both GPT-3.5 and Gemini-Pro across multiple parameters suggests that the model's underlying architecture and training processes have been significantly enhanced, enabling it to better mimic the reasoning and decision-making processes of human medical experts.

These findings are particularly noteworthy when considering the broader implications for AI in medical education. The model's high level of accuracy and logical consistency positions it not just as a tool for answering exam questions, but as a potential resource for developing personalized educational experiences that can adapt to the unique learning needs of individual students.

Potential applications and future directions

The results of this study suggest several promising applications for GPT-4 Turbo in medical education. One potential use case is as an adjunct to traditional learning methods, where the model can provide students with immediate feedback on practice questions, helping them to identify areas where they need further study. This could be particularly beneficial in preparation for high-stakes exams like the USMLE Step 1 or COMLEX Level 1, where precise knowledge and critical thinking are essential.

Another potential application is in the development of adaptive learning platforms that use AI to tailor educational content to the specific needs and learning styles of students. Gill et al. mention GPT-4 Turbo's ability to apply logical reasoning and incorporate external information into its responses, making it well-suited for this role, providing detailed explanations and contextual information that deepen students' understanding of complex medical concepts [28]. This could be explored through custom versions of ChatGPT specialized for medical education, an example seen in Grimoire, a GPT specialized for coding that is able to deliver specialized responses. 

Moreover, the adaptability of GPT-4 Turbo to new and unseen data suggests that it could be used to keep educational content up to date with the latest medical research and guidelines. This would ensure that students are always learning from the most current information, which is critical in a field as dynamic as medicine. Future research should explore these applications in more depth, particularly through longitudinal studies that assess the impact of AI-driven educational tools on student performance and knowledge retention. Additionally, there is a need to further refine models like Gemini-Pro to enhance their accuracy and logical reasoning capabilities, potentially expanding the range of tools available to medical educators.

Limitations

While this study highlights the impressive capabilities of GPT-4 Turbo, it is important to acknowledge its limitations. First, the model's performance, while superior, is not infallible. AlZu’bi et al. indicate the presence of logical and informational errors; although reduced, there is still room for improvement, particularly in ensuring that the model can consistently apply correct reasoning across a broader range of medical queries [29]. Second, the study primarily focused on the performance of these models on a specific set of NBME Step 1 questions. While this provides valuable insights into the models' capabilities, it also limits the generalizability of the findings to other types of medical exams or educational contexts. Further research is needed to evaluate the performance of these models across a wider range of medical topics and exam formats. Finally, while GPT-4 Turbo demonstrated strong adaptability to new data, the study did not explore how the model might perform in real-world clinical scenarios, where the complexity and variability of patient cases could present additional challenges. Future studies should aim to assess the model's performance in these contexts to better understand its potential applications and limitations in clinical practice.

## Conclusions

The results of this study suggest that GPT-4 Turbo represents a significant advancement in the use of AI for medical education. Its high accuracy, logical consistency, and adaptability to new data make it a promising tool for enhancing the learning experience of medical students. However, further development and testing are needed to fully realize its potential, particularly in ensuring that it can reliably support students in a wide range of educational and clinical scenarios. Gemini-Pro, while showing improvements over previous models, still faces challenges in achieving the same level of performance as GPT-4 Turbo. Nonetheless, with further refinement, it has the potential to become a valuable tool in the AI-driven educational landscape. As AI continues to evolve, its role in medical education is likely to expand, offering new opportunities to enhance learning outcomes and better prepare students for the demands of medical practice. This study contributes to the growing body of evidence supporting the use of AI in education and provides a foundation for future research aimed at optimizing these technologies for use in medicine.
